# Machine Learning Constructed Based on Patient Plaque and Clinical Features for Predicting Stent Malapposition: A Retrospective Study

**DOI:** 10.1002/clc.24332

**Published:** 2024-08-09

**Authors:** Qianhang Xia, Chancui Deng, Shuangya Yang, Ning Gu, Youcheng Shen, Bei Shi, Ranzun Zhao

**Affiliations:** ^1^ Department of Cardiology The Third Affiliated Hospital of Zunyi Medical University (The First People's Hospital of Zunyi) Zunyi China; ^2^ Department of Cardiology Affiliated Hospital of Zunyi Medical University Zunyi China

**Keywords:** acute myocardial infarction, machine learning, optical coherence tomography, percutaneous coronary intervention, stent malapposition

## Abstract

**Background:**

Stent malapposition (SM) following percutaneous coronary intervention (PCI) for myocardial infarction continues to present significant clinical challenges. In recent years, machine learning (ML) models have demonstrated potential in disease risk stratification and predictive modeling.

**Hypothesis:**

ML models based on optical coherence tomography (OCT) imaging, laboratory tests, and clinical characteristics can predict the occurrence of SM.

**Methods:**

We studied 337 patients from the Affiliated Hospital of Zunyi Medical University, China, who had PCI and coronary OCT from May to October 2023. We employed nested cross‐validation to partition patients into training and test sets. We developed five ML models: XGBoost, LR, RF, SVM, and NB based on calcification features. Performance was assessed using ROC curves. Lasso regression selected features from 46 clinical and 21 OCT imaging features, which were optimized with the five ML algorithms.

**Results:**

In the prediction model based on calcification features, the XGBoost model and SVM model exhibited higher AUC values. Lasso regression identified five key features from clinical and imaging data. After incorporating selected features into the model for optimization, the AUC values of all algorithmic models showed significant improvements. The XGBoost model demonstrated the highest calibration accuracy. SHAP values revealed that the top five ranked features influencing the XGBoost model were calcification length, age, coronary dissection, lipid angle, and troponin.

**Conclusion:**

ML models developed using plaque imaging features and clinical characteristics can predict the occurrence of SM. ML models based on clinical and imaging features exhibited better performance.

## Graphical abstract

This study investigated machine learning models to predict stent malapposition (SM) post‐PCI using OCT imaging, lab tests, and clinical data from 337 patients. Models, especially XGBoost, showed high accuracy, with key features being calcification length, age, coronary dissection, lipid angle, and troponin. Incorporating these features improved model performance significantly (*p* < 0.05).

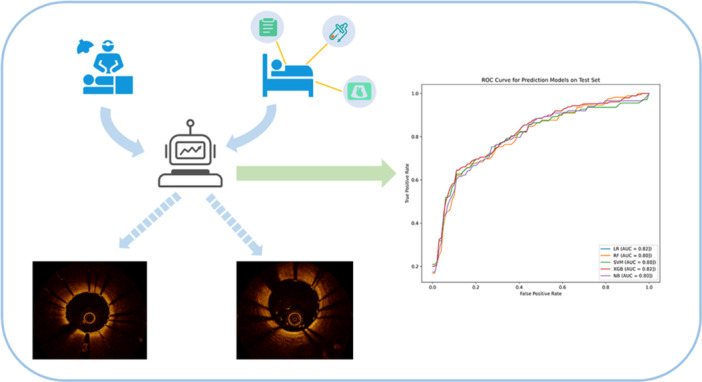

## 1 Background

Coronary artery disease (CAD) remains one of the leading causes of mortality and morbidity worldwide [1]. Acute myocardial infarction (AMI) represents one of its most severe manifestations. Percutaneous coronary intervention (PCI) is a commonly used treatment for CAD [2], which involves the percutaneous insertion of a catheter into the coronary artery, followed by the placement of a stent at the site of stenosis or occlusion to dilate the vessel and restore blood supply to the myocardium. However, PCI significantly improves the prognosis of CAD patients [3], and complications such as stent thrombosis [4] and in‐stent restenosis continue to pose significant challenges [5].

Among these complications, stent malapposition (SM), characterized by incomplete apposition of the stent, has emerged as a critical issue. SM may lead to thrombosis, restenosis, and even adverse events such as recurrent myocardial infarction [6, 7]. Therefore, identifying patients at risk of SM is crucial for optimizing clinical outcomes following PCI.

Traditional risk prediction models for SM are typically constructed based on clinical indices or angiographic features [8]. Nevertheless, these models often exhibit limited predictive accuracy and may fail to fully capture the complexity of patient‐specific factors influencing SM. In recent years, machine learning (ML) algorithms have shown potential in improving risk stratification and predictive modeling [9].

In this study, our aim is to explore the application of ML algorithms in predicting SM after PCI. By integrating data from multiple dimensions, including clinical registries, imaging databases, and electronic health records, we strive to develop a robust predictive model capable of identifying patients at high risk of SM. Specifically, we will focus on the application of optical coherence tomography (OCT) in this field. OCT, as a high‐resolution vascular imaging technique, can provide detailed information about stent position, intravascular morphology, and histological features [10]. In our predictive model, we will integrate plaque features obtained from OCT imaging with patients' clinical baseline data. By combining the quantitative analysis of OCT images with the pattern recognition capabilities of ML algorithms, we aim to establish a more precise SM prediction model. Such a model is expected to provide more accurate information for clinical decision‐making, assisting physicians in better assessing patient risk and devising more effective intervention strategies to reduce the occurrence of SM adverse events, thereby improving patient survival rates and quality of life.

## 2 Methods

### 2.1 Patient Data Selection

This is a retrospective observational study based on data collected from 337 patients who underwent PCI procedures at the Affiliated Hospital of Zunyi Medical University in China from May 2023 to October 2023. Among them, there were 279 male patients (82.8%) and 58 female patients (17.2%), with an average age of (61.5 ± 10.96) years. Inclusion criteria were as follows: (1) diagnosed with AMI, confirmed by coronary angiography to have at least one major coronary artery stenosis ≥ 75%, and received drug‐eluting stent PCI treatment; (2) underwent at least one OCT examination before and after the procedure; (3) complete clinical data available. Exclusion criteria were: (1) patients with lesions of restenosis after stenting; (2) patients with concomitant myocarditis, pericarditis, cardiomyopathy, congenital heart disease, valvular heart disease, and other organic heart diseases; (3) patients with poor image quality unsuitable for analysis. This study was approved by the Ethics Committee of the Affiliated Hospital of Zunyi Medical University.

### 2.2 PCI Procedure and Medication

All PCI procedures were performed by the same team of 2−3 experienced interventional cardiologists. Before emergency procedure, patients orally received enteric‐coated aspirin 300 mg and clopidogrel 300 mg. For elective procedures, patients were given aspirin 100 mg/day and clopidogrel 75 mg/day for 3 consecutive days before the procedure. During the procedure, patients were placed in a supine position, and the radial or femoral artery was punctured using the Seldinger technique. Subcutaneous infiltration anesthesia with lidocaine was used at the puncture site. Anticoagulation was achieved with 8000−10 000 U of heparin (depending on body weight), administered through the arterial sheath during the procedure. After surgery, subcutaneous injection of 4000 IU of low molecular weight heparin was given twice daily for 3−5 days, along with oral administration of aspirin 100 mg once daily and clopidogrel 75 mg once daily for at least 12 months. Treatment success was defined as residual luminal narrowing < 10% observed by visual assessment in at least two orthogonal projection views, achievement of TIMI 3 flow in the distal vessel beyond the stent, and absence of procedure‐related major complications (e.g., myocardial infarction, sudden death, and emergency CABG).

### 2.3 OCT Image Acquisition and Feature Analysis

Intravascular OCT imaging was performed using commercially available systems (ILUMIEN OPTIS, OPTIS Integrated, and OPTIS Mobile systems; Abbott Vascular), which include rapid‐exchange catheters (Dragonfly DUO, Dragonfly OPTIS, Dragonfly OpStar imaging catheters; Abbott Vascular) and an integrated pullback system (18−36 mm/s), acquiring blood displacement images with a high (~15 μm) axial resolution. Images were acquired as needed, after predilatation and administration of nitroglycerin within the coronary artery.

All OCT images were measured by two independent observers. In case of discrepancies between observers, consensus was reached regarding plaque measurements. Lipid plaques were defined as low‐signal regions with diffuse borders. Thin‐cap fibroatheroma (TCFA) was defined if the fibrous cap was < 65 μm thick, and the thinnest part of the fibrous cap contained lipid, with a lipid arc > 90°. Calcified plaques were defined as signal‐poor or heterogeneous areas with clear borders and a calcification arc ≥ 40°, without lipid plaques. Angles (°) were measured using a protractor centered on the lumen. SM was defined as at least one strut lacking contact with the vessel wall, with a distance of at least 150 μm [11].

### 2.4 Construction of ML Models

Current research suggests a close association between calcified plaques and SM [12]. To predict SM, we initially included five features: gender, age, calcified plaque length, calcified plaque angle, and calcified plaque thickness. We employed five ML models: Extreme Gradient Boosting (XGBoost), Logistic Regression (LR), Random Forest (RF), Support Vector Machine (SVM), and Naive Bayes (NB). Details of the specific parameters used for each algorithm in our analysis are provided in Supporting Information S1: Table 1.

Further optimization of the model involved performing Lasso regression to select features from clinical characteristics (16 baseline features, 24 laboratory examination features, 6 echocardiography features) and OCT plaque imaging features (21 features). The selected features were then reintegrated into the five ML algorithms mentioned previously for optimization. Specific parameters used in this optimization process are detailed in Supporting Information S1: Table 2.

### 2.5 Statistical Analysis

Kolmogorov−Smirnov tests were conducted to assess variable distributions. Continuous variables were expressed as mean ± standard deviation or median and interquartile range (IQR), and were compared using *t*‐tests or Mann−Whitney *U* tests as appropriate based on data distribution. Categorical variables were presented as counts and percentages and were compared using Fisher's exact test or chi‐square test as appropriate. Receiver operating characteristic (ROC) curves were plotted, and the area under the curve (AUC) was calculated to evaluate the predictive value of each model for SM. In addition, accuracy, sensitivity, specificity, positive predictive value (PPV), negative predictive value (NPV), F1 score, and optimal thresholds were calculated for each model. These metrics were computed as follows: sensitivity = true positive cases/(true positive cases + false negative cases), specificity = true negative cases/(true negative cases + false positive cases), PPV = true positive cases/(true positive cases + false positive cases), NPV = true negative cases/(true negative cases + false negative cases), accuracy = (true positive cases + true negative cases)/(true positive cases + false positive cases + true negative cases + false negative cases). Calibration curves were compared, and the calibration accuracy was assessed using the Hosmer−Lemeshow test. Statistical analysis was conducted using Python programming language version 3.9. *p* < 0.05 was considered statistically significant.

## 3 Results

**Figure 1:**
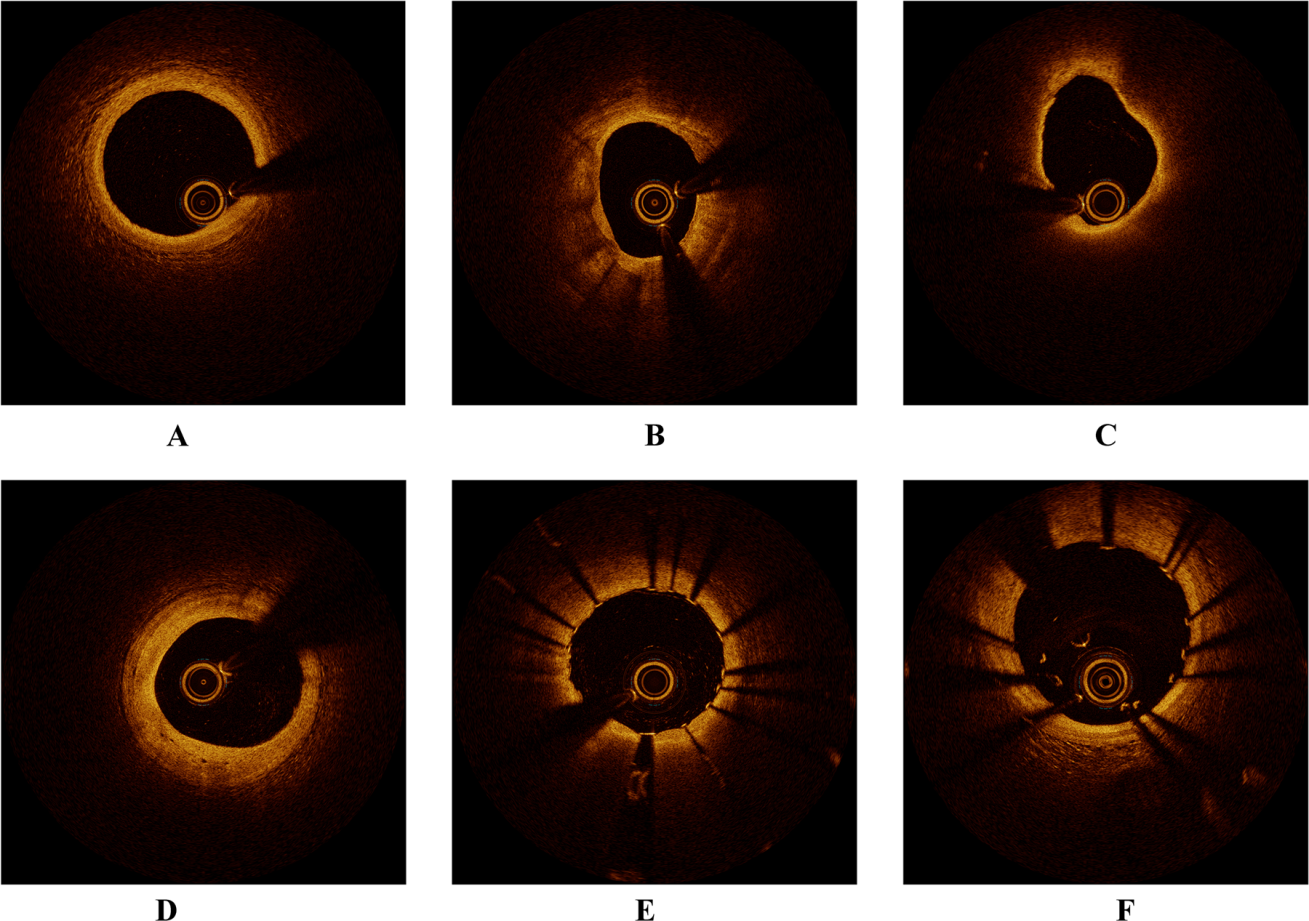
Partial OCT images. (A) Normal coronary OCT image; (B) calcified plaque; (C) lipid plaque; (D) microvessels; (E) non‐stent malapposition; (F) stent malapposition.

The final study included a total of 337 patients. The detailed patient selection flowchart is provided in Supporting Information S1: Figure 1. Examples of OCT images of partial lesions are shown in Figure 1. Clinical characteristics between the NSM and SM groups are presented in Table 1. There were no statistically significant differences between the two groups in terms of age, sex, and most laboratory parameters. However, the percentage of smokers in the SM group was higher than that in the NSM group (*p* = 0.038, Table 1). Imaging characteristics of all participants are listed in Table 2. Statistically significant differences between the two groups were observed in calcification length, calcification thickness, calcification angle, and calcification score (*p* < 0.001, Table 2). Additionally, there were differences between the two groups of patients in the presence of macrophage infiltration (*p* = 0.013, Table 2), microvessels (*p* = 0.014, Table 2), plaque erosion (*p* = 0.013, Table 2), and coronary dissection (*p* = 0.002, Table 2).

**Table 1:** Baseline characteristics.

	NSM (*n* = 227)	SM (*n* = 110)	*p*
Demographics			
Age (years)	62.00 [53.50−71.00]	59.00 [52.00−68.75]	0.140
Gender (%)			
Female	40 (17.6)	18 (16.4)	0.894
Male	187 (82.4)	92 (83.6)	
Weight (kg)	65.00 [61.00−70.00]	65.00 [60.00−70.00]	0.244
BMI (kg/m^2^)	25.34 [22.98−26.95]	24.13 [22.34−26.05]	0.169
Clinical features			
HR (BPM)	77.00 [70.00−87.00]	75.00 [68.50−84.00]	0.256
SBP (mmHg)	129.00 [113.50−143.00]	128.00 [113.25−140.00]	0.447
DBP (mmHg)	80.13 (13.17)	78.82 (12.61)	0.386
Hypertension (%)			
N	98 (43.2)	47 (42.7)	1.000
Y	129 (56.8)	63 (57.3)	
Diabetes (%)			
N	184 (81.1)	84 (76.4)	0.391
Y	43 (18.9)	26 (23.6)	
Hyperlipidemia (%)			
N	8 (3.5)	8 (7.3)	0.213
Y	219 (96.5)	102 (92.7)	
Smoker (%)			
N	87 (38.3)	56 (50.9)	**0.038**
Y	140 (61.7)	54 (49.1)	
CKD (%)			
N	226 (99.6)	108 (98.2)	0.520
Y	1 (0.4)	2 (1.8)	
UGIB (%)			
N	226 (99.6)	108 (98.2)	0.520
Y	1 (0.4)	2 (1.8)	
Af (%)			
N	224 (98.7)	108 (98.2)	1.000
Y	3 (1.3)	2 (1.8)	
Embolism (%)			
N	211 (93.0)	105 (95.5)	0.515
Y	16 (7.0)	5 (4.5)	
Laboratory data			
TG (mmol/L)	1.67 [1.13−2.69]	1.96 [1.26−2.65]	0.218
TC (mmol/L)	4.86 [4.14−5.53]	4.80 [3.92−5.53]	0.565
HDL‐C (mmol/L)	1.12 [0.97−1.29]	1.09 [0.94−1.22]	0.176
LDL‐C (mmol/L)	2.88 [2.34−3.45]	2.84 [2.10−3.45]	0.430
APOAI (g/L)	1.25 [1.15−1.35]	1.27 [1.13−1.34]	0.800
APOB (g/L)	0.96 ± 0.27	0.96 ± 0.30	0.942
Fasting glucose (mmol/L)	6.22 [4.83−8.62]	6.30 [5.30−8.23]	0.457
hs‐TNT (ng/L)	18.71 [8.07−156.00]	18.84 [8.07−223.38]	0.928
Myoglobin (ng/mL)	33.00 [23.00−64.50]	33.50 [21.00−71.25]	0.961
WBC count (10^9^ cells/L)	7.09 [5.74−9.12]	7.77 [6.00−9.22]	0.232
RBC count (10^9^ cells/L)	4.55 [4.20−5.04]	4.58 [4.14−4.92]	0.439
Platelet count (10^9^ cells/L)	202.00 [165.50−239.00]	205.00 [155.00−237.00]	0.848
ANC (10^9^ cells/L)	4.50 [3.46−6.30]	4.74 [3.64−6.45]	0.310
ALC (10^9^ cells/L)	1.56 [1.17−1.88]	1.50 [1.13−1.93]	0.880
AMC (10^9^ cells/L)	0.49 [0.37−0.67]	0.52 [0.37−0.70]	0.313
HGB (g/L)	140.00 [130.00−152.50]	139.00 [127.00−150.00]	0.262
ALT (U/L)	26.00 [19.00−37.00]	27.50 [18.00−43.75]	0.557
AST (U/L)	31.00 [24.00−49.50]	34.00 [23.00−54.00]	0.744
Urea (mmol/L)	5.40 [4.40−6.60]	5.40 [4.40−6.57]	0.979
Creatinine (umol/L)	77.00 [68.00−93.50]	76.50 [65.00−93.00]	0.537
eGRF (mL/min/1.73 m^2^)	83.90 [66.22−102.11]	85.16 [67.71−106.79]	0.341
BNP (pg/mL)	185.00 [71.00−628.50]	175.50 [72.50−700.00]	0.981
LDH (U/L)	229.00 [195.00−310.50]	232.00 [190.75−316.25]	0.791
HsCRP (mg/L)	3.47 [1.05−11.47]	2.74 [1.03−11.47]	0.219
Echocardiography			
LAd (mm)	33.00 [30.00−34.00]	33.00 [31.00−35.00]	0.642
IVSd (mm)	12.00 [10.00−12.00]	12 [10.00−12.00]	0.946
IVd (mm)	49.00 [46.00−52.00]	49.00 [45.00−51.75.00]	0.780
RVd (mm)	30.00 [29.00−32.00]	30.00 [28.00−32.00]	0.178
RAs (mm)	32.00 [30.00−33.00]	32.00 [30.00−33.00]	0.139
EF (%)	56.00 [50.00−61.00]	57.00 [52.00−62.00]	0.284

*Note*: The bold value indicates statistically significant result (*p* < 0.05).

Abbreviations: Af, atrial fibrillation; ALC, absolute lymphocyte count; ALT, alanine aminotransferase; AMC, absolute monocytes count; ANC, absolute neutrophil count; APOAI, apolipoprotein A‐I; APOB, apolipoprotein B; AST, aspartate aminotransferase; BMI, body mass index; BNP, B‐type natriuretic peptide; CKD, chronic kidney disease; DBP, diastolic blood pressure; eGRF, estimated glomerular filtration rate; HDL‐C, high‐density lipoprotein cholesterol; HGB, hemoglobin; HR, heart rate; HsCRP, high‐sensitivity C‐reactive protein; hs‐TNT, hypersensitive troponin; IVSd, interventricular septal thickness at diastole; Lad, left atrium diameter; LDH, lactate dehydrogenase; LDL‐C, low‐density lipoprotein cholesterol; LVEF, left ventricular ejection fraction; RAs, right atrial inner diameter; RBC, red blood cell; RVd, right ventricular internal dimension; SBP, systolic blood pressure; TC, serum total cholesterol; TG, triglycerides; UGIB, upper gastrointestinal bleeding; WBC, white blood cell.

**Table 2:** OCT features included in the statistical analysis.

	NSM (*n* = 227)	SM (*n* = 110)	*p*
Demographics			
Age (years)	62.00 [53.50−71.00]	59.00 [52.00−68.75]	0.140
Gender (%)			
Female	40 (17.6)	18 (16.4)	0.894
Male	187 (82.4)	92 (83.6)	
Location (%)			
LAD	153 (67.4)	70 (63.6)	0.622
LCX	21 (9.3)	9 (8.2)	
RCA	53 (23.3)	31 (28.2)	
Macrophages (%)			
N	127 (55.9)	45 (40.9)	**0.013**
Y	100 (44.1)	65 (59.1)	
Microvascular (%)			
N	143 (63.0)	53 (48.2)	**0.014**
Y	84 (37.0)	57 (51.8)	
Plaque rupture (%)			
N	165 (72.7)	80 (72.7)	1.000
Y	62 (27.3)	30 (27.3)	
Stratified plaque (%)			
N	188 (82.8)	86 (78.2)	0.382
Y	39 (17.2)	24 (21.8)	
Coronary dissection (%)			
N	194 (85.5)	78 (70.9)	**0.002**
Y	33 (14.5)	32 (29.1)	
Lipid plaque			
Lipid plaque fiber thickness (mm)	0.09 [0.06−0.14]	0.10 [0.06−0.18]	0.315
Lipid plaque arc (°)	216.80 [156.25−282.60]	229.00 [152.15−299.45]	0.469
Lipid plaque length (mm)	12.00 [8.50−17.25]	12.30 [8.12−17.20]	0.757
TCFA (%)			
N	128 (56.4)	67 (60.9)	0.502
Y	99 (43.6)	43 (39.1)	
Calcification plaque			
Calcified plaque (%)			
N	142 (62.6)	27 (24.5)	**< 0.001**
Y	85 (37.4)	83 (75.5)	
Calcification plaque thickness (mm)	0.00 [0.00−0.57]	0.66 [0.42−0.88]	**< 0.001**
Calcification plaque length (mm)	0.00 [0.00−3.55]	6.05 [2.95−9.20]	**< 0.001**
Calcification plaque arc (°)	0.00 [0.00−90.50]	128.60 [53.88−220.62]	**< 0.001**
Calcification plaque score (%)			
0	155 (68.3%)	27 (24.5)	**< 0.001**
1	32 (14.1）	15 (13.6)	
2	29 (12.8)	27 (24.5)	
3	3 (1.3)	6 (5.5)	
4	8 (3.5)	35 (31.8)	
Thrombus			
White thrombus (%)			
N	185 (81.5)	86 (78.2)	0.567
Y	42 (18.5)	24 (21.8)	
Red thrombus (%)			
N	191 (84.1)	90 (81.8)	0.703
Y	36 (15.9)	20 (18.2)	
Mixed thrombus (%)			
N	169 (74.4)	88 (80.0)	0.324
Y	58 (25.6)	22 (20.0)	

*Note:* Calcification plaque score: Maximum calcification thickness: < 0.5 mm—0 points; > 0.5 mm—1 point; maximum calcification length: ≤ 5 mm—0 points; > 5 mm—1 point; maximum calcification angle (°): ≤ 180°—0 points; > 180°—2 points. The bold values are indicate statistically significant results (*p* < 0.05).

Abbreviation: TCFA, thin‐cap fibroatheroma, defined as a lipidic plaque with the thinnest FCT < 65 μm and maximum lipid arc > 180°.

**Figure 2:**
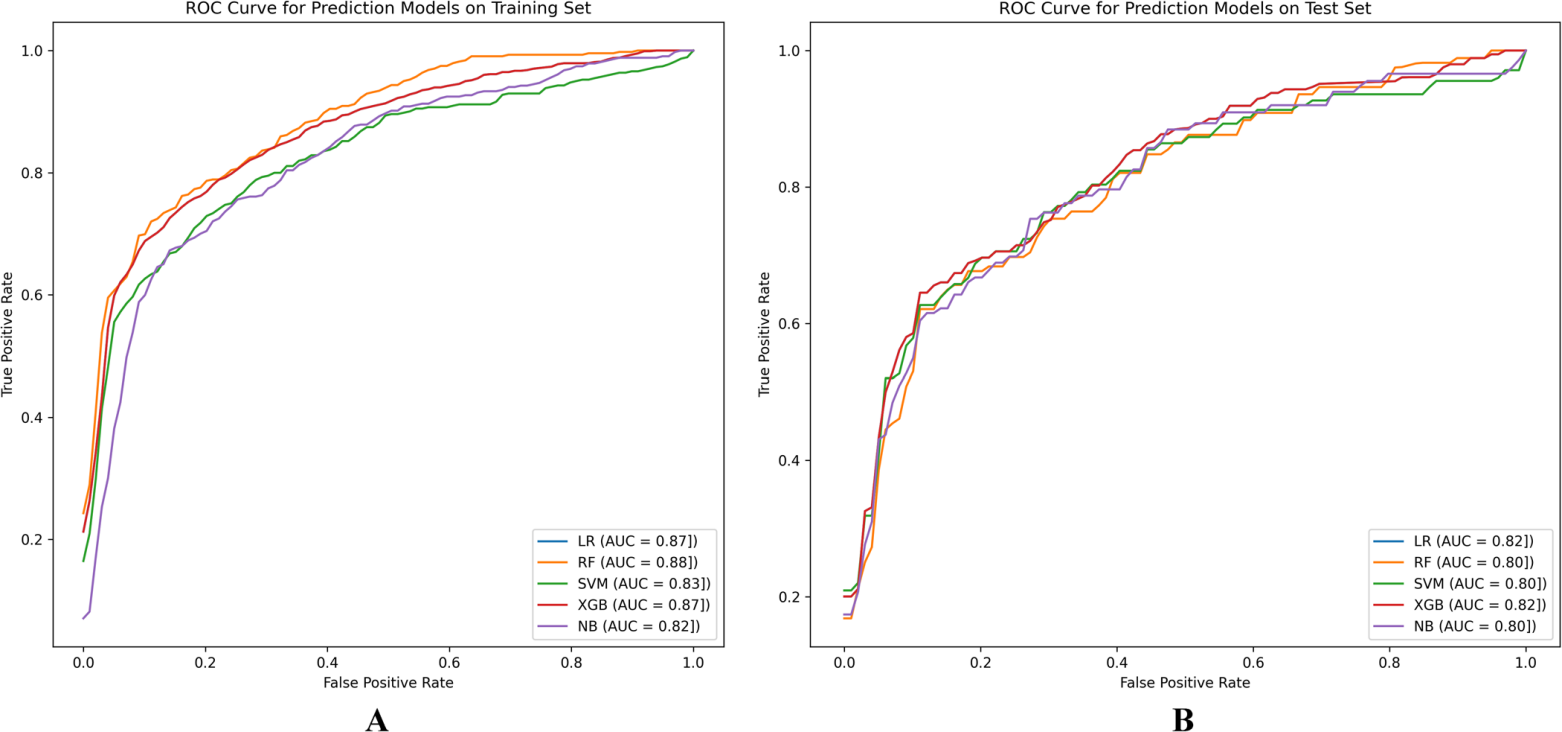
ROC curves of optimized machine learning prediction models for the training and testing data sets. (A) ROC curves of various algorithms for the training data set; (B) ROC curves of various algorithms for the testing data set.

We employed nested cross‐validation with fivefold cross‐validation to partition patients into training and validation sets for optimizing model parameters. After data preprocessing, a total of two baseline features (gender, age) and three plaque calcification‐related features (calcification plaque length, thickness, angle) were included for constructing five ML models. The performance of these models in predicting NSM and SM was evaluated using ROC curves for both the training set (Supporting Information S1: Figure 2A) and the test set (Supporting Information S1: Figure 2B). Compared to other models, the XGBoost and SVM models achieved higher AUC values in the test set (0.78, 0.78) (Supporting Information S1: Figure 2B).

**Figure 3:**
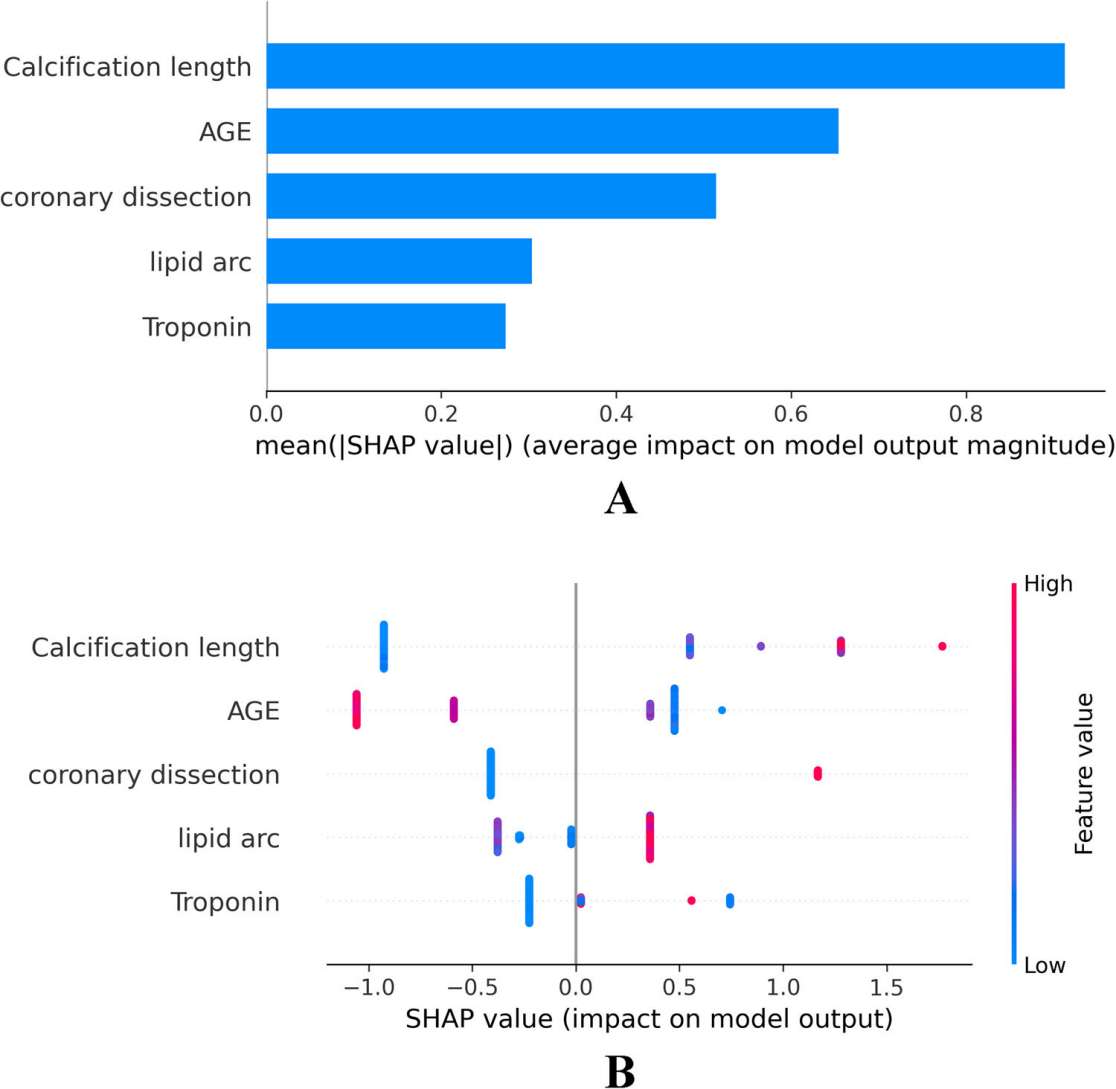
Feature importance of the XGBoost model assessed by SHAP value. (A) The influence of each feature on the output of the XGBoost model; (B) swarm plot.

A comprehensive summary of variables is presented in Tables 1 and 2. Initially, a total of 67 potential predictor variables were input into the feature selection process in the training set using fivefold cross‐validation. The dynamic process of the Least Absolute Shrinkage and Selection Operator (LASSO) and the relationship between the number of features and the minimum mean square error are shown in Supporting Information S1: Figure 3. Five key features were identified based on the minimum mean square error and values within 1 standard error, namely: lipid plaque angle, presence of microvessels, high‐sensitivity troponin, presence of coronary dissection, and smoking history (Supporting Information S1: Figure 3). These features were then incorporated into the model optimized with a focus on calcification features.

After incorporating the above features, the five ML algorithms were then employed. The performance of the optimized models in predicting NSM and SM is shown in Figure 2. All algorithms demonstrated improved predictive performance with higher AUC values in both the training set (Figure 2A) and test set (*p* < 0.05) (Figure 2B, Supporting Information S1: Table 3). After optimization, the AUC values of each model in the test set were closer, with the XGBoost and LR models achieving higher AUC values (0.82, 0.82) (Figure 2B). However, the XGBoost model achieved a higher F1 score (0.70, Supporting Information S1: Table 3). In terms of calibration analysis, the calibration curve of the XGBoost model was closer to the reference line compared to the LR model (Supporting Information S1: Figure 4). Furthermore, the Hosmer−Lemeshow test indicated a higher *p*‐value for the XGBoost model (0.774) compared to the LR model (0.146) (Supporting Information S1: Table 4). Therefore, the XGBoost model exhibited superior calibration in predicting outcomes than the LR model.

SHAP values reveal the feature rankings and the distribution of the impact of each feature on the output of the XGBoost model. The top five features were calcification length, age, coronary dissection, lipid angle, and high‐sensitivity troponin levels (Figure 3A). The swarm plot (Figure 3B) depicts high (red) and low (blue) values for numerical variables or “yes” (red) and “no” (blue) for binary variables.

## 4 Discussion

This study aimed to explore the predictive ability of ML models based on clinical features and calcified plaques for coronary SM in patients with AMI. SM is a common complication in the intervention of patients with AMI [13] and may lead to serious consequences, such as in‐stent restenosis [14], stent thrombosis [15], and even intravascular endothelial tears [16]. Early prediction of the risk of SM in patients is crucial for guiding clinical decisions and reducing adverse events [17]. Our study attempted to apply ML methods to this field by integrating clinical features and calcified plaque information to construct a predictive model for identifying the risk of SM in patients.

The study included 337 patients with AMI who underwent emergency PCI at our hospital. They all underwent pre‐ and postoperative OCT examinations. Most baseline characteristics such as gender and age showed no statistical differences between the two groups. However, the proportion of smoking patients in the SM group was higher than in the NSM group. Smoking is a well‐established cardiovascular risk factor. Although few studies directly link smoking to an increased incidence of SM post‐PCI [18], current research confirms that smoking has a close pathophysiological association with coronary plaque erosion [19].

In terms of OCT‐based plaque morphological features, there were statistically significant differences between the two groups in terms of calcification length, calcification thickness, calcification angle, and calcification score. This finding is consistent with the majority of current research [20, 21, 22]. Current studies indicate a close correlation between hard plaques, such as calcified plaques, and suboptimal stent apposition [23, 24]. However, there is relatively limited research on whether lipid‐rich plaques affect the occurrence of suboptimal stent apposition. In this study, there were no statistically significant differences between the two groups in terms of lipid plaque length, angle, and fibrous cap thickness (Table 2). There were statistically significant differences between the two groups of patients in the presence of macrophage infiltration (*p* = 0.013), microvessels (*p* = 0.014), and coronary dissection (*p* = 0.002) (Table 2). In coronary atherosclerotic lesions, inflammatory cell infiltration is closely associated with disease progression and plaque rupture [25, 26]. Macrophages and microvessels are important features for identifying vulnerable plaques [27]. These high‐risk plaques can lead to an increased incidence of adverse events post‐PCI [28].

The application of ML in the field of medicine has shown tremendous potential, particularly in predicting adverse events [29]. Traditional statistical methods often rely on specific assumptions and models, while ML algorithms are more flexible and capable of handling large‐scale, high‐dimensional data [30]. By learning from extensive clinical data, ML models can discover complex patterns and relationships within the data, thus providing more accurate predictions [31]. In ML, model interpretability is a crucial issue [32]. Particularly when using high‐dimensional data sets with numerous features, models may achieve high predictive accuracy, yet they can become complex and challenging to interpret. To address this problem, we initially included calcification‐related features that have been clearly associated with SM. Subsequently, we applied Lasso regression for variable selection to select key features, enhancing the clinical interpretability of the model. This method of stepwise feature inclusion helps effectively control the model's complexity, allowing us to construct a more streamlined and efficient model. Additionally, ML models are prone to overfitting, especially with smaller data sets [33]. To mitigate this issue, we employed stepwise feature inclusion and nested cross‐validation to improve the model's generalizability.

In this study, we employed five common ML methods and compared their performance in predicting SM in patients with AMI. Our results indicate that, in the test set, the XGBoost and SVM models based on calcification features achieved relatively high AUC values (AUC 0.78; Supporting Information S1: Figure 2). It is hypothesized that SM, as one of the potential adverse events of PCI, may not only be related to calcification but also involve other factors. To validate this hypothesis, we used Lasso regression for variable selection among 46 clinical features and 21 OCT‐based plaque features. Five key features were selected with values within one standard error of the minimum mean squared error (Supporting Information S1: Figure 3). These features include lipid plaque angle, presence of microvessels, high‐sensitivity troponin, presence of coronary dissection, and smoking history.

After incorporating these features into the five ML algorithms previously used for modeling, we observed improved AUC values for all five algorithms (*p* < 0.05) (Figure 2, Supporting Information S1: Table 3). Although all algorithms achieved AUC values above 0.8 in the test set and were relatively close, the XGBoost and LR algorithms exhibited slightly higher AUC values. This indirectly suggests that the occurrence of SM is not solely dependent on any single factor but rather the result of the combined effect of multiple clinical factors. However, the XGBoost model achieved a higher F1 score (0.70, Supporting Information S1: Table 3), and in the calibration analysis of the prediction model, the calibration curve of the XGBoost model was closer to the reference line (Supporting Information S1: Figure 4). The results of the Hosmer−Lemeshow test indicated a higher *p*‐value for the XGBoost model than the LR model (Supporting Information S1: Table 4). Therefore, the XGBoost model exhibited better calibration in predicting outcomes than the LR model. Thus, we conclude that for this data set, XGBoost outperforms the other models in terms of predictive performance. These results support the advantages of ML algorithms in handling multidimensional data due to their ability to process complex features, discover nonlinear relationships, and possess adaptability and generalization capabilities [34].

Based on the predictive model using the XGBoost algorithm, we further conducted SHAP analysis to explore the information gain (Figure 3). The results indicate that the top five features influencing the model are calcified plaque length, age, presence of coronary dissection, lipid angle, and high‐sensitivity troponin levels (Figure 3A). Current research supports the significant role of calcified plaques in the occurrence of SM [35, 36]. Although direct evidence linking patient age to the occurrence of SM is currently limited, numerous studies have confirmed a close association between age and the occurrence of coronary artery calcification [37, 38, 39]. In this study, the incidence of coronary dissection was higher in the SM group compared to the NSM group (*p* = 0.002). In the comprehensive feature analysis of the model, the occurrence of coronary dissection also significantly influenced the model predictions (Figure 3B). However, it is important to note that in this study, the coronary dissections were not spontaneous but rather Type A dissections formed after balloon predilation. Previous research indicates that approximately 40% of patients may experience dissections smaller than Type C after balloon angioplasty, which typically do not lead to acute coronary occlusion, but increase the risk of ischemia and patient mortality [40, 41]. With the advancement of intracoronary imaging, more instances of minor endothelial damage in coronary arteries are being observed [42]. Additional evidence from further studies is needed to determine whether these minor dissections contribute to adverse events following PCI procedures.

Although there were no statistically significant differences in features related to lipid plaques between the two groups of patients, the analysis of features in the comprehensive model reveals that lipid plaque angle has an important influence on the model output (Figure 3). These findings suggest that lipid plaques may potentially interact synergistically with calcified plaques, thereby increasing the occurrence of SM. However, further validation of this possibility is warranted by larger randomized controlled trials. In the baseline data analysis, there was no statistically significant difference in high‐sensitivity troponin levels between the two groups of patients (*p* = 0.928, Table 1). However, in the comprehensive feature analysis of the model, troponin levels also significantly contributed to the model outputs (Figure 3). Current research indicates that high‐sensitivity troponin measurement is not only a sensitive diagnostic method for myocardial infarction but is also associated with an increased risk of adverse events in myocardial infarction patients [43, 44].

Our study integrated coronary imaging and clinical features to develop a predictive model for predicting SM and NSM. By utilizing our developed predictive model, primary care physicians may potentially identify high‐risk patients before PCI, enabling personalized treatment plans. For patients predicted to be at high risk for SM, physicians can consider adjusting intraoperative strategies and selecting more suitable stent types or surgical approaches. Primary care physicians dealing with complex cases can use ML models as auxiliary decision‐making tools. The predictive results and feature analyses provided by the model can offer additional information support, aiding them in making more accurate clinical decisions.

This study integrated coronary imaging features and clinical characteristics to establish a comprehensive predictive model. This multidimensional feature integration enhanced the predictive capability of the model. We used multiple advanced ML algorithms and evaluated their performance using ROC curves. We improved model interpretability by employing stepwise feature inclusion. Additionally, nested cross‐validation was used to randomly allocate training and validation sets, thus reducing the risk of overfitting.

In addition, there are several limitations to this study. First, our model is based on a retrospective cohort study, which may introduce selection bias. Additionally, being a single‐center study, it lacks an external validation cohort. This study solely evaluates the occurrence of SM and does not assess whether these apposition anomalies are associated with adverse events. Future prospective studies involving multiple medical centers will be necessary to validate the universality and reproducibility of our model.

## 5 Conclusion

In summary, we developed and validated a novel ML model, specifically the XGBoost model, for predicting SM by integrating plaque imaging features and clinical characteristics. The XGBoost model demonstrated superior performance with higher AUC values and calibration compared to other models. Our study demonstrates the potential of AI and ML in predicting SM, helping clinicians make optimal decisions to improve patient outcomes.

## Conflicts of Interest

The authors declare no conflicts of interest.

## Supporting information

Supporting information.

## Data Availability

The data that support the findings of this study are available from the corresponding author upon reasonable request.
