# Supplementary material for: Machine Learning Constructed Based on Patient Plaque and Clinical Features for Predicting Stent Malapposition: A Retrospective Study
Source: Clin Cardiol. 2024 Aug 9;47(8):e24332. doi: 10.1002/clc.24332 (PMC11310765; doi:10.1002/clc.24332)
Supplement: Supplementary file 1 — Supporting information. [file CLC-47-e24332-s001.docx]

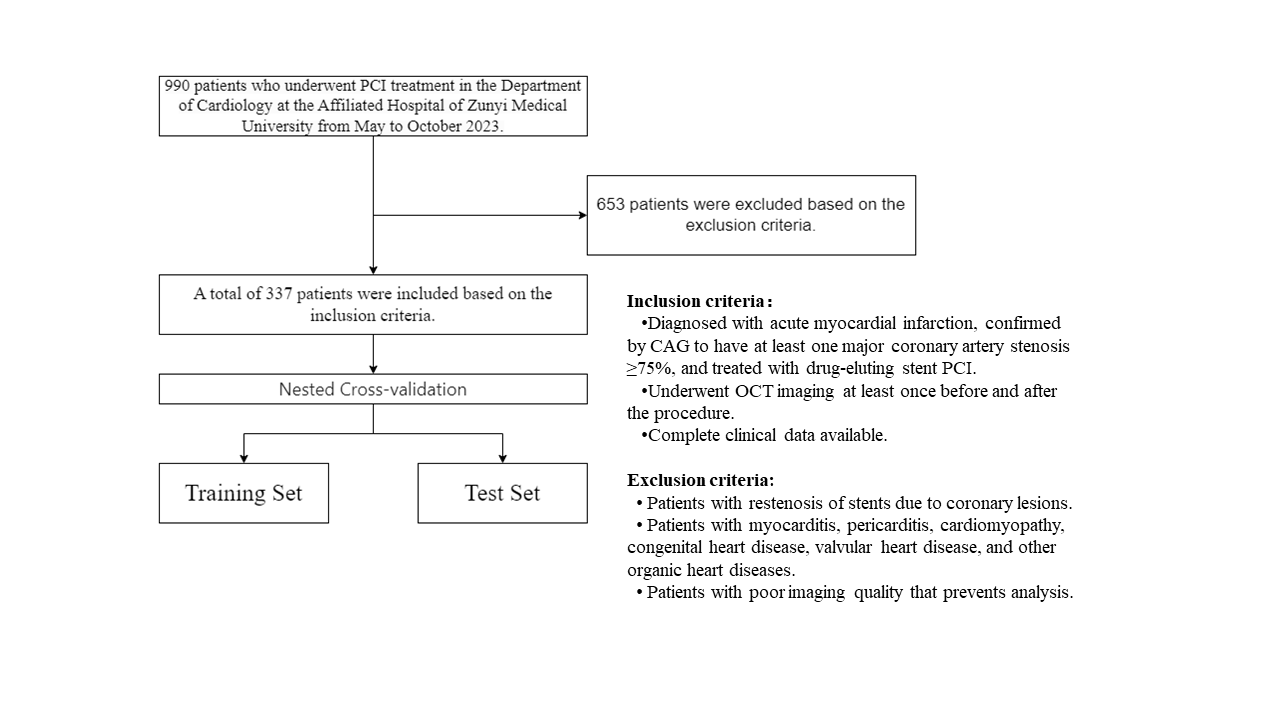


Supplementary Figure 1: Inclusion and exclusion criteria for patient


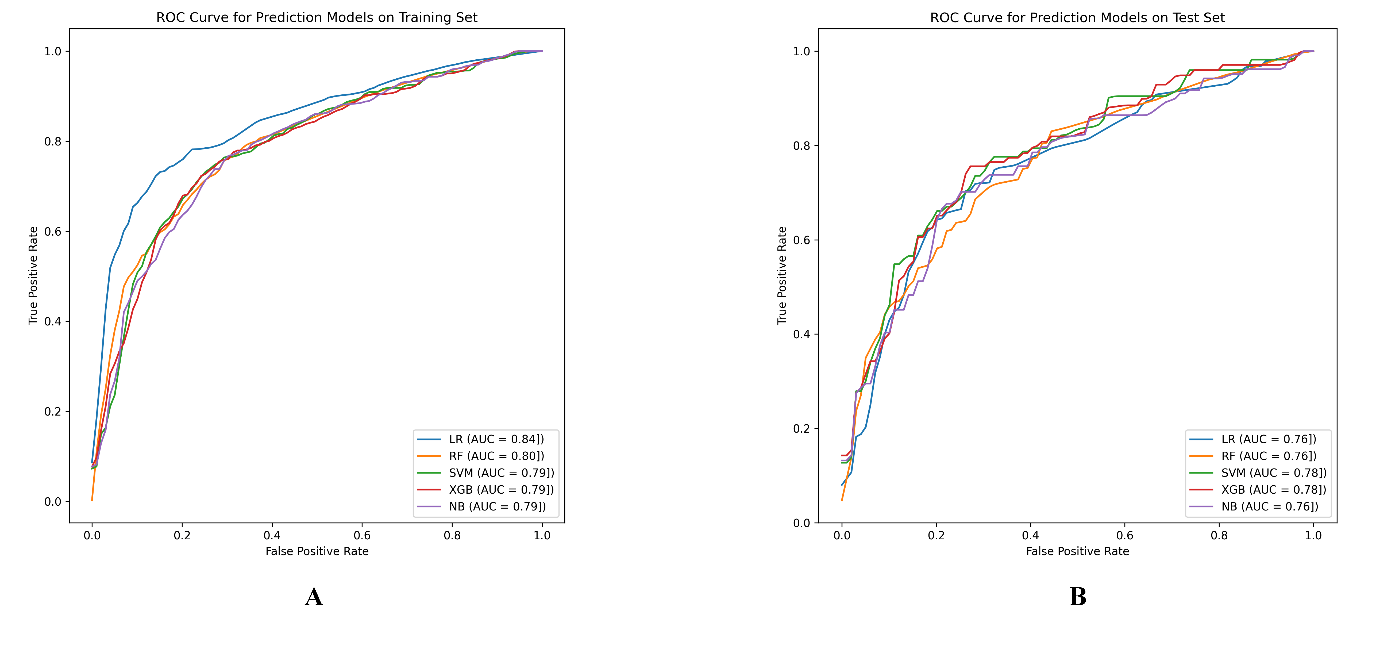


Supplementary Figure 2: ROC curves of machine learning prediction models based on calcification plaque features for the training and testing datasets. A) ROC curves of various algorithms for the training dataset; B) ROC curves of various algorithms for the testing dataset.


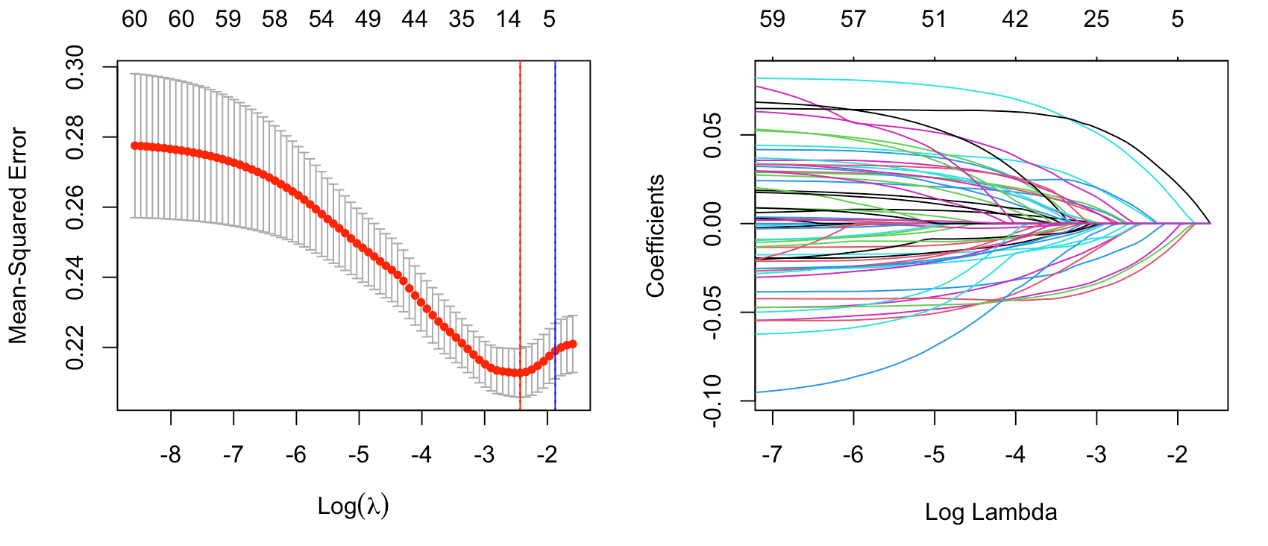


Supplementary Figure 3: Feature selection based on LASSO regression.


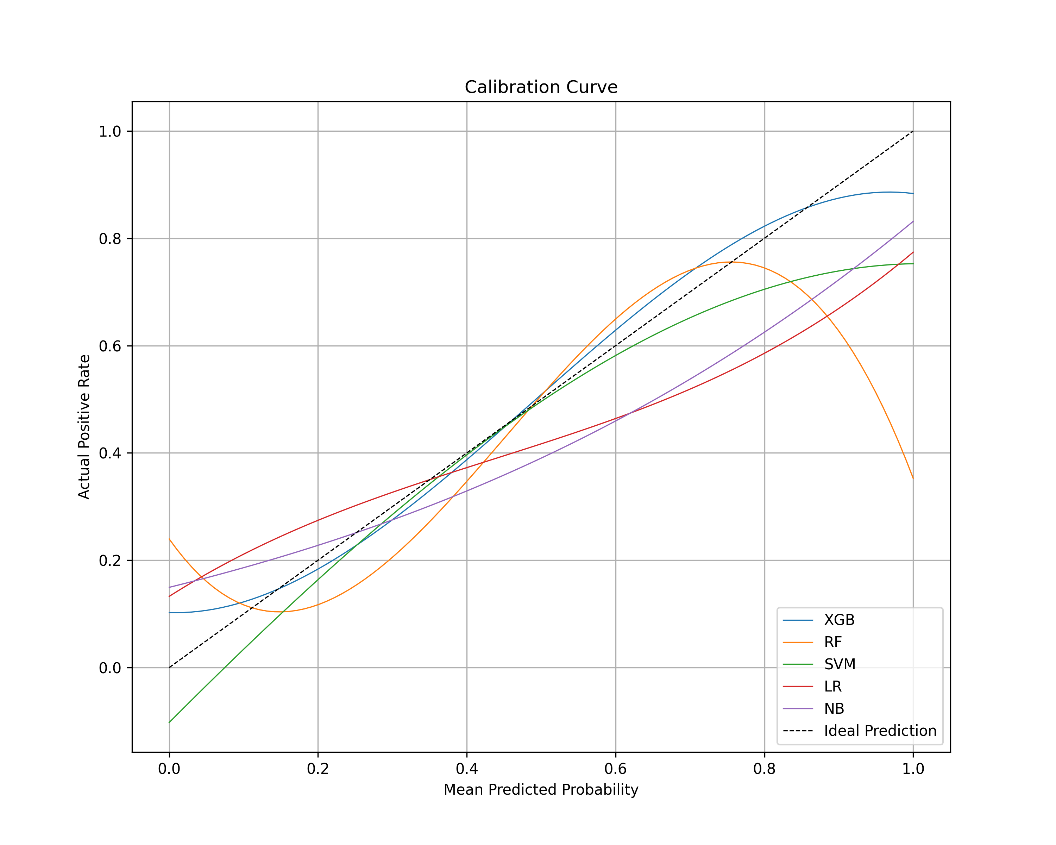


Supplementary Figure 4: Calibration curves of the predictive models.

Supplementary Table 1: Five algorithms based on calcification features and their parameters:

| Model | parameters |
| --- | --- |
| XGBoost | n_estimators=30, scale_pos_weight=2.06, gamma=1, max_depth=2 |
| LR | solver='liblinear', C=2 |
| RF | n_estimators=42, max_depth=1 |
| SVM | kernel='linear', C=0.2 |
| NB | priors=None |

LR：Logistic Regression; RF：Random Forest; SVM: Support Vector Machine; NB: Naive Bayes.

Supplementary Table 2: Five optimized algorithms based on multi-dimensional features and their parameters:

| Model | parameters |
| --- | --- |
| XGBoost | n_estimators=73, gamma=1, max_depth=1, scale_pos_weight=2.06 |
| LR | solver='liblinear', C=0.3 |
| RF | n_estimators=50, max_depth=3, min_samples_split=0.4 |
| SVM | kernel=' linear ', C=1, probability=True |
| NB | priors=None |

LR: Logistic Regression; RF: Random Forest; SVM: Support Vector Machine; NB: Naive Bayes.

Supplementary Table 3: Performance evaluation of optimized algorithms:

| Model | XGBoost | SVM | RF | NB | LR |
| --- | --- | --- | --- | --- | --- |
| Optimal thresholds | 0.70 | 0.55 | 0.74 | 0.39 | 0.34 |
| AUC（95%Cl） | 0.82(0.78, 0.86) | 0.80 (0.76, 0.84) | 0.80(0.75 - 0.84) | 0.80(0.76,0.84) | 0.82(0.77,0.85) |
| AUC *p*-value | 0.002 | 0.037 | 0.004 | 0.002 | 0.002 |
| SEN（95%Cl） | 0.64(0.46, 0.72) | 0.29(0.09, 0.46) | 0.64(0.38, 0.89) | 0.37 (0.12,0.64) | 0.55(0.33,.075) |
| SPE（95%Cl） | 0.91 (0.82, 0.94) | 0.95(0.89, 0.99) | 0.84(0.75,0.93) | 0.95(0.89,0.99) | 0.91(0.83,0.97) |
| ACC（95%Cl） | 0.82(0.77, 0.89) | 0.82(0.71, 0.88) | 0.81(0.72,0.89) | 0.83(0.75,0.92) | 0.82(0.75,0.90) |
| PPV（95%Cl） | 0.78(0.62, 0.92) | 0.57(0.15, 0.94) | 0.5(0.27,0.74) | 0.63(0.25,0.97) | 0.63(0.41,0.85) |
| NPV（95%Cl） | 0.84(0.75, 0.92) | 0.85(0.75,0.90) | 0.91(0.81,0.98) | 0.86(0.77,0.94) | 0.87(0.79,0.95) |
| F1 score（95%Cl） | 0.70(0.59, 0.73) | 0.38(0.11,0.57) | 0.56(0.32,0.76) | 0.45(0.15,0.70) | 0.59(0.39,0.74) |

LR: Logistic Regression; RF: Random Forest; SVM: Support Vector Machine; NB: Naive Bayes; AUC: Area Under the Curve; AUC *P*-value: Compared with the AUC of the model before optimization; SEN: Sensitivity; SPE: Specificity; ACC: Accuracy; PPV: Positive Predictive Value; NPV: Negative Predictive Value.

**Supplementary Table 4.** **Hosmer-Lemeshow test results for each model**

| Model | XGBoost | SVM | RF | NB | LR |
| --- | --- | --- | --- | --- | --- |
| Hosmer-Lemeshow *P*-value | 0.774 | 0.031 | 0.064 | 0.001 | 0.146 |

LR: Logistic Regression; RF: Random Forest; SVM: Support Vector Machine; NB: Naive Bayes.
